# Association of rodents with man-made infrastructures and food waste in Urban Singapore

**DOI:** 10.1080/20008686.2021.2016560

**Published:** 2022-01-28

**Authors:** Hui Yun Penny Oh, Mahathir Humaidi, Qian Yi Chan, Grace Yap, Kai Yang Ang, Jason Tan, Lee Ching Ng, Diyar Mailepessov

**Affiliations:** a Environmental Health Institute, National Environment Agency, Singapore, Singapore; b Environmental Public Health Operations Department, National Environment Agency, Singapore, Singapore; c School of Biological Sciences, Nanyang Technological University, Singapore, Singapore

**Keywords:** *Rattus norvegicus*, rat infestation, bin chambers, rat tracking, residential estates

## Abstract

**Background:**

Rodent population control is an important measure in reducing the risk of rodent-borne disease transmission. In this study, we examined rodent activity in the sanitary waste network around the household waste-collection bin chamber of an urban residential apartment block.

**Methods:**

We utilised infra-red camera traps to determine the pattern of rodent activity in a rodent-infested bin chamber and its associated sanitary waste network. Multivariable logistic regression was performed to assess the risk factors that were independently associated with rodent activity in the bin chambers.

**Result:**

The camera trap surveillance showed that the rodents were active in the bin chamber and sanitary network both in the day and at night. In the cross-sectional study, rodent activity in the bin chambers was independently associated with broken floor traps [Adjusted odds ratio (AOR): 36.7, CI: 21.3–66.3], calendar month [Log-likelihood ratio test (LRT) *p* = 0.002] and Town Council [LRT *p* = 0.004] variables. In restricted analysis, rodent activity in bin chambers was independently associated with defects in the wastewater pipe under the chamber [AOR: 12.3, CI: 4.3–51.7].

**Conclusion:**

Our study suggests that urban municipal management councils should prioritize rodent control resources in areas according to the factors that increase the risk of rodent infestation.

## Introduction

Rodents are successful vectors for many zoonotic pathogens that cause significant morbidity and mortality in humans. Rodent-borne diseases are prevalent in all continents except Antarctica with a higher number of outbreaks reported in America, Asia, and Europe [1]. To date, over 70 known rodent-borne diseases have been reported thus far [2]. While it is known that around 60% of the human cases of infectious disease are acquired from animals, the true burden and prevalence of rodent-borne diseases have not been defined globally due to inadequate surveillance and absence of uniform reporting system for many of the diseases [3]. Leptospirosis is one such example. Despite being coined as the most widespread rodent-borne zoonotic disease, with 1.03 million cases of infection and 58,900 deaths annually, it is highlighted as a neglected zoonotic disease by the World Health Organisation [4,5].

The majority of the invasive rat species are known to originate from Asia and over the years, humans have inadvertently assisted in their colonization of the world [6–8]. *Rattus norvegicus* and *Rattus rattus* are the two most common invasive rat species typically found in almost all continents [9–11]. They are known to be highly resilient and can adapt to live in almost all environments. Environmental factors such as adequate harbourage and ease of access to food and water, makes certain areas more prone to infestation [12]. In urban centres, *Rattus norvegicus* burgeons in habitats with ample food waste and favourable burrowing sites such as sewer and underground subways [13–15]. *Rattus rattus*, on the other hand, prefers to seek refuge in the upper levels of buildings, both nesting close to human settlements [15,16]. It is projected by 2050, 68% of the world population will reside in the urban environment [17]. The urban rats are known to reach sexual maturation earlier as compared to their rural counterparts [18]. Coupled with ineffective rodent control measures and their proximity to humans, problems of sporadic rat–human contact and disease transmission will only escalate in the future. The urban environment, if poorly managed, is subject to a confluence of factors that can provide rodents easy access to food and harbourage [19]. In addition to the increase in public health risks, these urban rats are notorious for depredating of food for human consumption and compromising infrastructure [20]. However, control measures taken against persistent infestations have often led to substantial economic losses [20,21]. Given the unprecedented rates of global urbanization, it is crucial to better understand how to manage rodents in urban centres.

A repertoire of rodent control techniques have been developed over the years, and they can be broadly divided into three main categories, namely, biological, chemical, and physical controls [22]. Chemical and physical methods such as the use of rodenticides and traps account for over 70% of the rodent control techniques used worldwide, while biological techniques such as the deployment of natural predators (*Felis domestica*) and parasites (*Sarcosystis singaporensis*) to control rodent populations are less widely used, as pest management professionals have found the former to be effective and cost-efficient [22–27]. Infrastructural designs and materials are used to exclude rodents although no study has been conducted to evaluate the effectiveness of these different designs [28–30]. Despite the diverse range of control and exclusion techniques, there is no effective solution to completely eradicate the rodents owing to the inherent challenges and limitations of the methods as well as the poor understanding of rodent behaviour and ecology [24–26,31–35].

The presence of illegal dumping sites and infrequent garbage clearance are often correlated with the rise in rodent populations and infestations by invasive rodent species [36–40]. Hence, efficient removal of municipal solid waste to curb their access to food waste is an important aspect to manage rodents, particularly in a densely populated urban environment. In this study, we investigated rodent activity in the bin chambers of an urban residential apartment block and assessed the risk factors for rodent activity in these bin chambers at the national level.

## Material and methods

### Study area

Public residential apartment buildings built by the government are home to over 80% of the residents in Singapore [41]. The remaining population resides in landed houses and condominiums built by private developers. The Town Councils discharge the responsibilities of estate management in public residential estates, such as but not limited to the cleaning of common areas, building maintenance, horticultural landscaping, estate improvement works, vector control, and municipal waste collection. The municipal waste collection frequency in public housing estates is typically once or twice daily, depending on waste volume (including weekends and statutory public holidays) [42]. The National Environment Agency (NEA) has three regional offices (west, east, and central) that routinely conduct ground surveillance and audits for rodent control, in public areas that include the common areas of these housing estates. Previous observations have noted the association of rodent infestation with waste system in many of these blocks of apartments. In blocks built prior to 1989, the Individual Refuse Chute System (IRCS) (Supplementary Figure 1) equipped each unit of apartment with a hopper and a refuse bin in a chamber at the end of the chute on the ground floor. A removable plastic refuse bin collects all the refuse disposed of from the residential units that are served by the chute. The refuse bin can be accessed via a metal door whereby workers empty the refuse bin contents manually. The graded tiled floor in the refuse bin chamber allows any sullage to flow into the sanitary lines via a floor trap in the chamber.

A public residential block with seven IRCS located in the eastern part of Singapore was selected as the first study site (**Appendix 1**). The residential block is known to have a perennial rodent infestation issue in its refuse bin chambers. We visually inspected all seven chambers and observed that rats escaped through broken floor traps upon disturbance. To understand the rodent ecology beneath and around the bin chamber, we selected the most infested IRC chamber (target bin chamber) to monitor for rat activity within the bin chamber as well as the sanitary-line network that was shared across all seven chutes.

### Study Design

Field observational study was conducted over March and April 2016 to investigate rodent activity and movement patterns around the waste bin chamber. The second component was a national-level cross-sectional study conducted between August 2017 and April 2018 to analyse risk factors associated with rodent activity across all public housing estates.

## Phase 1 study design

**Camera traps for the detection and recording of rodent activity**

Rodent activity in the sanitary drainage system and the IRCS was monitored using a Reconyx PC900 Professional Covert Camera Trap (Reconyx, USA). This camera is equipped with passive infra-red (PIR) motion and heat sensor arrays, which are activated when a warm object passes in front of the PIR array. The camera trap also features a 0.2 s trigger speed, which makes it suitable for capturing rodent activity. With a top running speed of 3.6 m/s, a rodent can cover 0.72 m after it has entered the PIR trigger zone. This allows the camera to capture the images of any rodent that enters its field of view.

The camera trap was set to capture three successive images with every trigger. A trigger with at least one of its three images showing rodent presence was considered as a positive trigger. Conversely, a trigger with no rodent image was considered as a false trigger. To quantify the activity level across time, we divided an hour into 12 five-minute activity blocks. An activity block is defined by the activation of at least one positive trigger within a span of five minutes. In an hour, the rodent activity count will range between 0 and 12 five-minute activity blocks.

**Preliminary monitoring of rodent activity in target bin chamber and connecting sanitary line**

For the preliminary monitoring of rat activity, camera traps were deployed in the target waste bin chamber and the direct-connecting inspection chamber (DCIC) of the sanitary line leading to the chamber, from 1100 to 2200 hours (Supplementary Figure 2). For the bin chamber, a camera trap was placed on the floor facing the floor trap and in the DCIC, it was secured magnetically under the DCIC cover. Recorded images were analysed for rodent activity levels over 11 hours.

**Monitoring of rodent activity within the associated sanitary-line network**

Camera traps were installed magnetically and activated at 1800 hours underneath the seven sanitary-line inspection chamber (IC) covers and the DCIC cover of the target bin chamber. The DCIC, ICs and target bin chamber were left undisturbed for 2 hours to allow for the rodents to acclimatize to the cameras and allow sufficient time for the rodents to resume their feeding activity. Rodent activity was captured by the camera traps overnight from 2000 hours till 0700 hours.

### Inspection of floor trap pipe integrity

The Olympus Series C Videoscope no. IV0620C (Olympus Corporation) was used to inspect the inner surfaces of floor trap pipes. The videoscope has an articulated camera at the end of a 1.5 m steel braided flexible hose. The floor trap pipe of the target bin chamber was inspected, and the recorded video was visually analysed to ascertain the structural integrity of the pipe and signs of rodent presence, such as fresh droppings or live rats.

## Phase 2 study design

**Risk factors for rodent activity in public housing estates**

Island-wide surveillance of bin chambers was conducted concurrently across the five community development districts demarcated in Singapore, namely the Central, North-East, North-West, South-East, and South-West districts between August 2017 and April 2018 (duration of ~8 months). NEA also conducted an extensive bi-monthly Island-wide rodent burrow surveillance, and areas with 20 or more burrows were classified as ‘Red Clusters’. The top-five ranking Red Clusters in terms of burrow counts were flagged out as Red Clusters of Concern (RCC) monthly. A list of old public housing blocks with more than 25 years of age (i.e., constructed before 1989 as of mid-2017) was gathered for reference prior to the surveillance, which was sequentially carried out by constituency delineated within each community development district for ease of data collation. Environmental health officers inspected every bin chamber (n = 5,002) located at the foot of the block within an 8-month duration for structural lapses (e.g., defective floor traps, gully traps, broken floor tiles, corroded bin chamber door, and ledge) and signs of rat activity (e.g., droppings, live rats, and dead rats). Bin chambers were assessed to have rat activity or rat infestation if they exhibited one or a combination of the following traits: Droppings in the bin chamber and/or on the recessed area in front of the chamber, live and dead rat sightings. Following the Island-wide surveillance of bin chambers, those with defective floor traps (n = 230) were sieved for inspection of sub-structural defects with the use of a videoscope. Sub-structural defects largely consist of broken sanitary pipelines visible only at the subterranean level, which could be readily exploited by rats underground to gain immediate access to food sources in bin chambers. As sub-structural defects may extend deep into the subterranean level and may, therefore, be erroneously excluded from the collations, the presence of soil in sanitary lines alluding to implications from rat activity was used as a proxy for the possible presence of an underground sub-structural defect. Due to the time lapse between the original Island-wide surveillance and videoscope inspection, any changes in structural conditions resulting from intervention by stakeholders (e.g., bin chamber repairs) were accounted for accordingly in the collated data list.

### Statistical analysis

We investigated the association between the rodent infestation in the bin chambers and broken floor trap, using multivariable logistic regression model adjusted for potential confounders. The dependent variable ‘rodent infestation’ was assigned to be 1 when the presence of rodent activity was detected, as described above, or 0 otherwise. The main independent variable ‘broken floor trap’ was assigned to be 1 when the integrity of the floor trap was compromised or 0 otherwise. ‘Month’ variable was coded as the categorical variable denoting the month of the year when the bin chamber was inspected. ‘Town Council’ variable was coded as the categorical variable denoting which town council takes charge of the area where observation was collected. ‘RCC’ was coded as 1 when the inspected bin chamber was located within the area of the RCC cluster and 0 otherwise. ‘Regional office’ was coded as a categorical variable denoting, which regional offices take charge of the area where the observations were recorded.

A multivariate logistic regression model based on association between rodent infestation and broken floor trap adjusted for potential confounders was built in several steps. First, we created a core model consisting of the dependent variable ‘rodent infestation’ and the main independent variable ‘broken floor trap’. We then tested the influence of other independent variables on the association by adding that variable to the core model. The log-likelihood ratio test (LRT) was then conducted to compare the core model and the model with the added variable. All variables for which the LRT *p*-value was lower than 0.05 were considered to be statistically significant and were selected for the full model. The LRT test was then used to justify the retention of the variable from the full model in the final model. Full model was compared to the sub-model that included all variables of the full model, except the variable tested. After testing all the variables in the same manner, variables that were statistically significant were included in the final model. Statistical analyses were performed using R version 3.6.1.

For the videoscope study, a multivariable logistic regression model was used to investigate the association between the presence of rodents in bin chambers and the presence of sub-structural defects, adjusted for potential confounders. The dependent variable, similarly to the island-wide study, was set to be the rodent infestation in the bin chamber, coded as 1 if the bin chamber was infested or 0 otherwise. The main independent variable, presence of sub-structural defects coded as 1, and 0 when there was no defect. The ‘Month’, ‘Town Council’, and ‘regional office’ variables were coded the same way as in the Island-wide study. ‘RCC’ data was not collected for the videoscope study, while ‘floor trap’ categorical variable was further stratified into ‘defective’, ‘normal’ or ‘absent’.

The step-wise procedure of achieving the most parsimonious model was similar to what was described for the island-wide study. The core model was built first investigating the association between rodent infestation and the presence of sub-structural defects underneath the floor trap. Then, the influence of each variable on this association was tested by adding the variable to the core model and conducting LRT with the new and the core models. Variables whose influence was found to be statistically significant in the LRT test (*p-*value <0.05) were then added to the full model. The retention of the variables from the full to the final model was again justified using the LRT. Statistical analyses were performed using R version 3.6.1.

## Results

Observation of rat activity around a bin chamber

**Figure 1.**
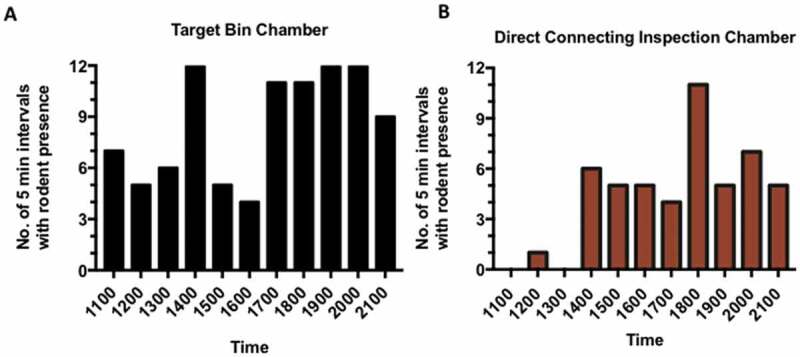
Observations of rodent activity in individual bin chamber and direct connecting inspection chamber. The number of 5-minute intervals with rodent presence from 1100 to 2200 hours for (**A**) individual refuse chute chamber and (**B**) direct connecting inspection chamber.

In the rodent activity monitoring study, the camera trap recorded 1,046 triggers in the bin chamber and 285 triggers in the DCIC from the time 1100 hours to 2200 hours. The rats were active in the day and we noted a heightened activity during 1400 to1500 hours and after 1700 hours (Figure 1).

**Figure 2.**
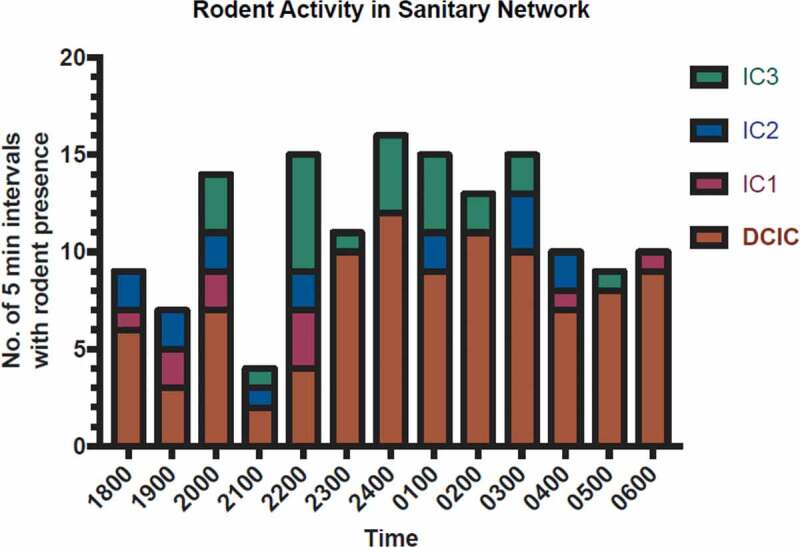
Rodent activity in direct connecting inspection chamber (DCIC) and inspection chambers (ICs) with a summary table showing total number of triggers caused by rodents. The number of 5-minute intervals with rodent presence was plotted from 1800 to 0700 hours the next day.

For the sanitary network monitoring, we observed high rodent activity in the DCIC overnight for a 12-hour period (Figure 2). Rat activity was also detected in increasing order in IC1, IC2, and IC3, while no rat activity was detected in IC4 to IC7. IC 1, 2 and 3, each spaced 14 m apart, are in decreasing distance to the target bin chamber and are of depths 1 m, 1.2 m and 1.4 m, respectively. The target bin chamber, DCIC, and IC3 are each spaced 3 m apart (**Appendix 1**). Our videoscope footages uncovered that the floor trap pipe of the target bin chamber was compromised, and we noted the presence of a sub-structural defect (void space) right next to the opening of the compromised pipe (Supplementary Figure 2A). Our videoscope footage also captured the presence of live rats in the compromised floor trap pipe (Supplementary Figure 2B). Our data suggested that rats were active throughout the studied time in the infested bin chamber and the associated sanitary network with the highest rodent activity was found nearer to the target bin chamber. Our data also suggested that the compromised floor trap and sub-structural defect provided the rats easy access to travel between the bin chamber and the ICs.

Our national-level cross-sectional study revealed that the odds of rodent activity in bin chambers were much higher when the floor trap was broken (AOR: 36.7, CI: 21.3–66.3, *p* < 0.001) (Table 1). Rodent activity was higher in the months of October and November compared to December (AOR: 8.1, CI: 2.5–29.0, *p* < 0.001, AOR: 4.2, CI: 1.7–11.5, *p* < 0.001). There were statistically significant differences in rodent activities among Town Councils using Aljunied-Hougang as the reference category (see Table 1). Rodent activity was also higher in chambers found within the RCC (AOR: 3.2, CI: 1.4–7.1, *p* < 0.005). Finally, the odds of rodent activity were higher in bin chambers with sub-structural defects (AOR: 12.3, CI: 4.3–51.8, *p* < 0.001) (Table 2).

**Table 1.** Multivariable logistic regression analysis of factors collected in Island-wide bin chamber survey associated with rodent infestation. For the ease of interpretation, the reference strata were selected to display positive associations for statistically significant AOR. Variables with p < 0.05 are highlighted in **bold**. CI stands for confidence interval

	Rodent infestation			
	Adj odds ratios	CI	*p*	*LRT*
Broken floor trap	36.7	21.3–66.3	<0.001	<0.001
Months				0.002
January	1.4	0.5–4.5	0.530	
February	2.2	0.7–7.4	0.205	
March	2.2	0.6–8.4	0.258	
August	0.8	0.0–5.6	0.853	
September	1.2	0.3–4.3	0.777	
October	8.1	2.5–29.0	0.001	
November	4.2	1.7–11.5	0.003	
December	Ref			
Town Council				0.004
Ang Mo Kio	7.3	1.4–58.9	0.033	
Bishan-Toa Payoh	4.8	0.5–46.0	0.156	
Choa Chu Kang	1.4	0.3–11.1	0.696	
East Coast	1.8	0.3–14.7	0.540	
Holland-Bukit Timah	2.6	0.3–25.2	0.380	
Jalan Besar	3.0	0.1–34.8	0.394	
Jurong-Clementi	12.6	3.0–88.4	0.002	
Marine Parade	6.9	1.8–46.7	0.015	
Marsiling-Yew Tee	2.9	0.1–33.9	0.409	
Nee Soon	2.7	0.6–19.5	0.259	
Pasir Ris-Punggol	0.0	0.00–0.00	0.988	
Sembawang	9.1	0.4–107.1	0.086	
Tampines	7.8	1.3–65.9	0.033	
Tanjong Pagar	6.0	0.6–60.0	0.107	
West Coast	22.5	3.9–189.8	0.001	
Aljunied-Hougang	Ref			
RCC	3.2	1.4–7.1	0.005	0.007

**Table 2.** Multivariable logistic regression analysis of factors collected in videoscope study associated with rodent infestation. Variables with p < 0.05 are highlighted in **bold**. CI stands for confidence interval

	Rodent infestation		
	*Odds ratios*	*CI*	*P*
Defective sub-structure	12.3	4.3–51.7	<0.001

## Discussion

In Singapore, rodent burrows can be found near residential estates and near bin chambers and bin centres. Despite having an enclosed system for municipal waste disposal, rodents are a common sight around the refuse bins, and this poses potential health risks for the nearby residents and waste collectors. To date, no study has been conducted to understand how rodents access the municipal waste system. A better understanding of their spatial ecology will hence provide useful insights for targeted eradication of the long-established rat population in Singapore.

Our findings shed light on the ecology of rats around the refuse bins. Footages from the videoscope revealed that the floor trap pipe and the surrounding sub-structure were compromised, and rats were harbouring within the sub-structure beneath the target bin chamber. Movements of rats are mostly detected in the harbourage under the bin chamber.

The presence of live rodents in the sub-structural defect space linked to the defective floor trap pipe suggests that rodents might have capitalized on the compromised sub-structure, caused by either mechanical wear and tear or active rodent gnawing, as a potential harbourage point. Due to the length limitation of the videoscope, we were unable to probe into the sub-structure that extends beyond 1.5 meters. We did not observe any nesting sites for the rodents. Therefore, we suspect that the whole burrow network might extend further underground, and this can be further investigated in a future study.

Our nationwide cross-sectional study has also identified several factors associated with rodent infestations in the municipal waste system of Singapore (e.g., broken floor trap, presence of defective sub-structure beneath the bin chamber, RCC, Month, and town council). Though the identified factors are novel and/or have not been well characterized in Singapore, they agree with well-known environmental factors that favour rodent infestations with some examples including the presence of suitable harbourage and the ease of access to food and water, which are noted to be strong drivers for rodent infestations [43,44].

Surface rat infestations in the urban environment have always been thought to arise from the underground and that surface populations reflect those in the underground [45]. However, our nationwide study showed that more than 99% of the well-maintained refuse chambers (functional floor trap and no structural defects), including those from high surface burrow count areas, were not rodent infested. Due to the manual nature of waste removal, rodents from the surface are also able to breach into the bin chamber, while the worker is clearing the bin or when the bin chamber door is not closed properly after bin clearance. This might also explain why RCC tends to have rodent infestation in the bin chambers. Being highly adaptable and mobile creatures, we infer that rodent infestations in the refuse bin chamber may originate from either the substructure or from the surface, and successful harbourage depends on the presence of malfunctioning rodent proofing features or practices.

We would also like to highlight that it is still plausible for rats to leave the sanitary lines and bin chamber in an event of overcrowding [46]. Furthermore, the levels of rodent infestation were different among different management councils, possibly due to differences in pest management practices employed by various pest control contractors, and the varying tolerance level of residents towards rodent presence [47]. The odds of infestation were higher in the months of October and November compared to December. This could be due to seasonality in the rodent activity, however, which needs to be studied more in-depth.

In conclusion, we have identified several risk factors that are associated with rodent infestations in the municipal waste system of Singapore. We have also highlighted the importance of having a functioning floor trap cover to prevent rodents’ access to food waste. Targeted complete eradication of the existing underground rodents followed by routine surveillance to ensure proper maintenance of floor traps and underlying sub-structures in the bin chambers around residential estates will offer a long-term solution to curb rodent infestation.

## Data Availability

Data supporting the conclusions of this article are provided within the article. The datasets used and/or analysed during the present study are available from the corresponding author upon reasonable request.

## Appendix 1

Layout showing the study site of interest. The target refuse bin chamber (red star) with internal and external defects within the HDB estate (mustard) is linked to the direct connecting inspection chamber (DCIC in green) and inspection chambers (IC1-7 in maroon) as shown. These inspection chambers are built to allow underground drainage pipes to be routinely inspected and managed by the TC. Liquid waste from the target refuse bin chamber is discharged into the underground municipal sewer as shown.
